# A Transcriptomic Atlas Underlying Developmental Plasticity of Seasonal Forms of *Bicyclus anynana* Butterflies

**DOI:** 10.1093/molbev/msac126

**Published:** 2022-06-09

**Authors:** Shen Tian, Antónia Monteiro

**Affiliations:** Department of Biological Sciences, National University of Singapore, Singapore, Singapore; Department of Biological Sciences, National University of Singapore, Singapore, Singapore

**Keywords:** phenotypic plasticity, developmental plasticity, seasonal polyphenism, alternative splicing, miRNA, post-transcriptional regulations

## Abstract

Organisms residing in regions with alternating seasons often develop different phenotypes, or forms, in each season. These forms are often adaptations to each season and result from an altered developmental response to specific environmental cues such as temperature. Although multiple studies have examined form-specific gene expression profiles in a diversity of species, little is known about how environments and developmental transitions, cued by hormone pulses, alter post-transcriptional patterns. In this study, we examine how gene expression, alternative splicing, and miRNA-mediated gene silencing in *Bicyclus anynana* butterfly hindwing tissue, varies across two rearing temperatures at four developmental timepoints. These timepoints flank two temperature-sensitive periods that coincide with two pulses of the insect hormone 20E. Our results suggest that developmental transitions, coincident with 20E pulses, elicit a greater impact on all these transcriptomic patterns than rearing temperatures per se. More similar transcriptomic patterns are observed pre-20E pulses than those observed post-20E pulses. We also found functionally distinct sets of differentially expressed genes (DEGs) and differentially spliced genes (DSGs) in the seasonal forms. Furthermore, around 10% of DEGs are predicted to be direct targets of, and regulated by, differentially expressed miRNAs (DEmiRs) between the seasonal forms. Many DEGs, DEmiRs, or DSGs potentially regulate eyespot size plasticity, and we validated the differential splicing pattern of one such gene, *daughterless*. We present a comprehensive and interactive transcriptomic atlas of the hindwing tissue of both seasonal forms of *B. anynana* throughout development, a model organism of seasonal plasticity.

## Introduction

Some organisms can change their physiology, behavior, or morphology in response to different environmental cues, an ability referred to as phenotypic plasticity. Plasticity can often be adaptive because different phenotypes are usually better suited for different environments (Stearns 1989; West-Eberhard 1989). Classical examples of phenotypic plasticity include predator-induced helmets in water fleas, changes in the shape of leaves in response to water submersion, changes in social insect caste in response to nutrition, and changes in butterfly wing patterns in response to seasons (Pfennig et al. 2010; Beldade et al. 2011). However, how such plastic systems have evolved at the molecular level to allow the environment to modulate the process of development, to generate different phenotypes from the same genome, is still poorly understood.

Emerging evidence suggests that both differential gene expression as well as less well-studied post-transcriptional processes such as alternative splicing, RNA editing, and micro-RNA (miRNA)-mediated gene silencing, all impact plastic traits (Marden 2008; Gommans et al. 2009; Li et al. 2014). Many studies have examined the differential gene expression patterns between different morphs in a series of polyphenic species (Brisson et al. 2007; Chen et al. 2012; Jiang et al. 2012; Daniels et al. 2014; Vilcinskas and Vogel 2016). Other studies have also revealed alternative splicing profiles in flies, pea aphids, fishes, and plants in response to different environmental cues (Long et al. 2013; Jakšić and Schlötterer 2016; Shang et al. 2017; Grantham and Brisson 2018; Healy and Schulte 2019). Differential alternative splicing is also observed in different castes of eusocial insects such as ants and bees (Foret et al. 2012; Cingolani et al. 2013; Li-Byarlay et al. 2013). The roles of miRNAs in mediating phenotypic plasticity have only begun to be elucidated. MiRNAs are small, noncoding RNAs of 20–22nt in length that usually bind 3′ untranslated regions (3′UTRs) of messenger RNAs (mRNAs) to mediate mRNA decay (Filipowicz 2005; Bartel 2009). Recent RNA deep sequencing projects revealed a large number of miRNAs differentially expressed in several polyphenic insects, including pea aphids, locusts, and butterflies (Wei et al. 2009; Legeai et al. 2010; Mukherjee et al. 2020). In addition, miRNAs are also essential regulators of insect hormonal signaling involving 20-hydroxyecdysone (20E) biosynthesis, *Ecdysone (20E) receptor* (*EcR*) expression, and the expression of primary 20E response genes (Varghese and Cohen 2007; Jiang et al. 2013; Xiong et al. 2016; He et al. 2017, 2019; Peng et al. 2019).

**Fig. 1.**
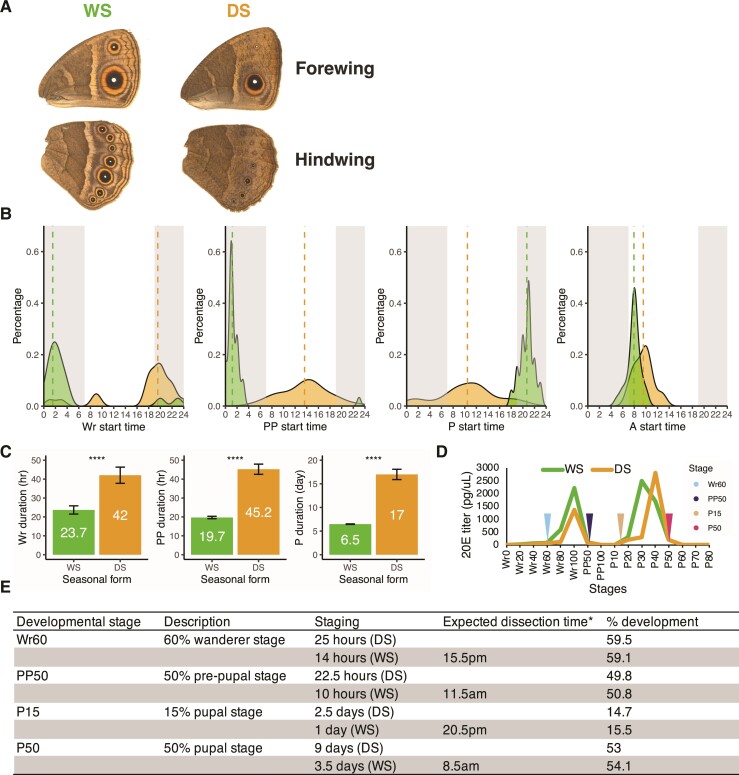
Staging of *B. anynana* seasonal forms for transcriptomic analysis. (*A*) Wet season (WS) and dry season (DS) forms of *B. anynana* butterflies exhibit different hindwing eyespot sizes. (*B*) Population-level distribution of the start time (time of day) and (*C*) duration of the wanderer (Wr) stage, prepupal (PP) stage, pupal (P) stage, and adult (A) stage in the two seasonal forms. Each dataset includes measurements from *N* individuals (*N* = 20–41). Dotted lines mark the mean values of the start time. Shaded area indicates night, whereas unshaded area indicates daytime. Error bar represents standard deviation. Statistical significance was assessed via Student’s *t*-test. ns, not significant; *, *P* < 0.05; **, *P* < 0.01; ***, *P* < 0.001; ****, *P* < 0.0001. (*D*) Hindwings in both seasonal forms were collected at 60% Wr stage (Wr60), 50% PP stage (PP50), 15% P stage (P15), and 50% pupal stage (P50) for RNA-seq and sRNA-seq (black arrowheads). Hormone titers were measured previously (Monteiro et al. 2015). (*E*) Detailed staging criteria. The staging h/days describe time since the beginning of the Wr, PP, or P stage. The expected dissection times (*) for the WS individuals were the same as their actual dissection times, whereas the expected dissection times calculated from the population level for the DS samples were of less relevance since the developmental transition start times of DS individuals were weakly coordinated to photoperiod. The expected dissection times of DS samples were precisely determined for each DS individual, and DS samples were dissected only if their expected dissection times were comparable to those of the WS samples (see Materials and Methods for more detail).

To unravel how both gene expression as well as post-transcriptional processes are regulated during development in a plastic system, we focus on the African satyrid butterfly, *Bicyclus anynana*. This has been a classic model species for studies of phenotypic plasticity, primarily in wing color patterns. Some of its populations live in regions with alternating seasons, where changes in temperature cue the arrival of different future selective environments, and lead to the development of different forms of the butterfly (Brakefield and Reitsma 1991). The wet season (WS) form, which develops at high rearing temperature, exhibits large ventral hindwing eyespots, whereas the dry season (DS) form, that develops at lower temperature, exhibits very small eyespots (Brakefield and Larsen 1984; Brakefield and Reitsma 1991) (fig. 1*A*). Besides the plastic eyespot size, DS forms are also darker, with less contrasting wing color patterns, such as the transversal white band, the golden ring, and the eyespot centers (fig. 1*A*) (Mateus et al. 2014; Monteiro et al. 2015; van Bergen and Beldade 2019). The current hypothesis is that perhaps two different guilds of predators, with more invertebrate predators present in the WS, shape these wings patterns to either be prominent and conspicuous, to help deflect attacks toward the wing margin, or small and cryptic to prevent detection all together (Lyytinen et al. 2004; Prudic et al. 2015). Taken together, irrespective of the ultimate selective pressures that shape these alternative wing patterns, *B. anynana* has evolved a mechanism, cued by temperature, to develop these alternative forms, using the same genome.

One of the early mechanisms explored for the regulation of wing pattern plasticity in *B. anynana* was the endocrine system. Endocrine systems are often involved in the process of translating environmental cues to distinct phenotypes in polyphenic species (Dufty et al. 2002; Nijhout 2003). In *B. anynana*, higher rearing temperatures lead both to higher 20E hormone titers in a pulse during the wandering (Wr) stage, and to an earlier rise of a second 20E pulse in the pupal stage of WS forms relative to DS forms (fig. 1*D*) (Oostra et al. 2011; Monteiro et al. 2015). These distinct hormone profiles, coupled with the expression of the 20E receptor, *EcR*, in the hindwing eyespots of both forms, contribute to regulate hindwing eyespot size plasticity in this species (Mateus et al. 2014; Monteiro et al. 2015).

The 20E hormone system, however, is unlikely to be the only mechanism translating temperature to alternative phenotypes in *B. anynana*. It is possible that other hormonal systems or temperature-sensitive but endocrine-independent signaling pathways also contribute to the regulation of wing color pattern plasticity. This is because manipulations of 20E signaling alone, either at the Wr stage or early pupal stage, are insufficient to produce complete seasonal form mimics (Mateus et al. 2014; Monteiro et al. 2015).

Few studies have attempted to examine the interplay between hormone-mediated development and environment. In the case of *B. anynana*, how developmental transitions mediated by 20E pulses, and rearing temperatures, collaboratively remodel the landscape of various transcriptomic patterns, and how they correlate with each other, is still unknown. To make further progress on the molecular mechanisms of seasonal plasticity, we performed an unbiased transcriptomic analysis to decipher the genome-wide omics patterns in the seasonal forms of *B. anynana*. We conducted RNA-sequencing (RNA-seq) and small RNA-seq (sRNA-seq) on female hindwings of both seasonal forms across four developmental timepoints just before and after the larval and pupal 20E pulses (fig. 1*D*). Since we are particularly interested in the regulation of eyespot size plasticity in the seasonal forms, we sequenced hindwing tissues, as hindwings have more eyespots and their eyespots are more plastic than those on the forewings (fig. 1*A*) (Monteiro et al. 2015). Three transcriptomic patterns consisting of (1) gene expression, (2) alternative splicing, and (3) miRNA-mediated gene silencing, and their correlations, were assessed in both seasonal forms throughout wing development.

## Results

### Staging of *B. anynana* Seasonal Forms

Precise staging of development is essential to compare seasonal forms at equivalent stages, especially when the seasonal forms show a different pace of development. Only females were used in the current study to avoid dealing with variation across sexes. Starting times of the wanderer (Wr) stage, prepupal (PP) stage, pupal (P) stage, and adult (A) stage (fig. 1*B*), and durations of the Wr, PP, and P stages (fig. 1*C*), were measured in both seasonal forms. In general, the starting times when individuals transit from one stage to the next, are highly gated with regards to photoperiod in WS forms, whereas those of DS forms appear more dispersed. Adult (A) emergences happen at around the same time of the day in both seasonal forms, but the starting times of Wr, PP, and P stages do not overlap between seasonal forms (fig. 1*B*). Durations of Wr, PP, and P stages are all significantly prolonged in DS forms (fig. 1*C*). Based on the previously measured 20E titers in both seasonal forms (fig. 1*D*) (Monteiro et al. 2015), female hindwings were dissected according to the staging measurements (fig. 1*E*) at 60% Wr stage (Wr60), 50% PP stage (PP50), 15% P stage (P15), and 50% P stage (P50), since they are critical developmental timepoints when late larval and pupal 20E titers start to rise or drop to basal levels (fig. 1*D*).

Note that although great efforts have been made to sample seasonal forms from the equivalent stages, it is possible that some of the seasonal form changes we were trying to capture could still be attributed to small discrepancies in developmental stage or other factors. This is inevitable. However, we assume that most changes in omics patterns observed from DS and WS samples from the equivalent stages are differences induced by rearing temperatures, the only variable we changed to induce the two seasonal forms. Also, it is also likely that not all the omics pattern differences observed between seasonal forms will contribute to morphological differences in adult butterflies but may instead represent a general physiological response to different rearing temperatures.

### Developmental Transitions Cued by 20E Pulses have a Larger Impact in Remodeling Gene Expression and Alternative Splicing than Rearing Temperatures

**Fig. 2.**
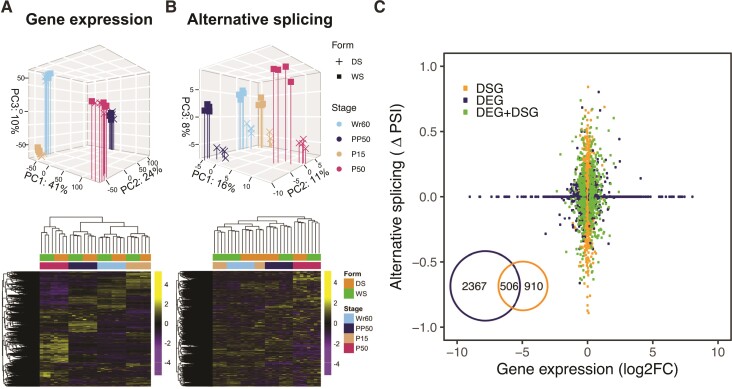
Global gene expression and splicing patterns suggest the presence of distinct sets of DEGs and DSGs between seasonal forms. (*A*) Genome-wide gene expression and (*B*) alternative splicing patterns in the seasonal forms across four developmental timepoints were assessed using PCA and hierarchical clustering heatmaps using all genes. (*C*) The scatter plot shows the magnitudes of gene expression differences (log2FC) of DEGs (adjusted *P* value [*P*adj] < 0.05), and inclusion level differences (ΔPSI) of DSGs (False Discovery Rate [FDR] < 0.05), between seasonal forms (WS form vs. DS form) during Wr60. Venn plot shows the number of DEGs, DSGs, and genes belonging to both sets. The other developmental timepoints are shown in supplementary figure S1, Supplementary Material online.

Genome-wide profiles of gene expression and alternative splicing were assessed across all data sets. Gene expression and splicing similarities were analyzed via principal component analysis (PCA) and hierarchical clustering trees. In the PCA of gene expression, the first three major PCs (cumulatively 75% of total variance) separate the four developmental stages (fig. 2*A*, upper). In the hierarchical clustering tree, gene expression is primarily clustered by developmental stage, then by seasonal form (fig. 2*A*, lower). This indicates that developmental transitions trigger a substantially larger shift in gene expression than rearing temperatures do. In addition, Wr60 and P15, the two stages before the larval and pupal 20E pulses, respectively, are more closely clustered than PP50 and P50, the two stages after the larval and pupal 20E pulses, respectively (fig. 2*A*, lower). This indicates that each 20E pulse may induce dramatic- and stage-specific changes in gene expression in larval and pupal wings.

For alternative splicing, we first assessed the number of alternative splicing events in each sequencing library and across all libraries using rMATS (Shen et al. 2014). rMATS detects five types of alternative splicing events: skipped exons (SE), alternative 5′ splice sites (A5SS), alternative 3′ splice sites (A3SS), mutually exclusive exons (MXE), and retained introns (RI). Overall, 23,169 alternative splicing events were detected in 4,270 genes across all sequencing libraries. Around 36% of all expressed genes (Transcripts Per Million [TPM] > 0.1) had at least one alternative splicing event across all sequencing libraries (supplementary table S1, Supplementary Material online). Regarding different types of splicing event, a higher proportion of MXE and SE sites were detected relative to the other types from each sequencing library (except MXE found in WS_P50), and across all libraries (supplementary table S2, Supplementary Material online).

We then examined the global alternative splicing pattern across the sequencing libraries. In the PCA of alternative splicing, PC1 and PC2 (cumulatively 27% of total variance) separate the four developmental stages, whereas PC3 (8% of total variance) separates seasonal forms (fig. 2*B*, upper). In the hierarchical clustering tree, alternative splicing is primarily clustered by seasonal forms and then by developmental stages during Wr60 and P15, but the opposite happened during PP50 and P50, post-20E pulses (fig. 2*B*, lower). This suggests that temperature plays a primary role in remodeling alternative splicing patterns pre-20E pulses, whereas development plays the dominant role post-20E pulses. As observed for gene expression, Wr60 and P15 are also more closely clustered than PP50 and P50 (fig. 2*B*, lower). This suggests that the 20E pulses during larval and pupal stages, which have different intensities and dynamics in the two seasonal forms, not only impact gene expression, but also affect alternative splicing in a stage-specific manner.

### Seasonal Forms Exhibit Functionally Distinct Sets of Differentially Expressed Genes and Differentially Spliced Genes

We then asked whether the differentially expressed (DE) genes (DEGs) were also differentially spliced (DS) genes (DSGs) between seasonal forms, or whether the DEGs and DSGs belonged to two different sets. To examine this, we first created sets of DEGs and DSGs for each developmental stage, and then examined the correlation between these gene sets between seasonal forms. Around 1,697–2,873 DEGs (*P*adj < 0.05) were discovered between seasonal forms at each developmental stage (supplementary tables S3 and S4, Supplementary Material online). For DSGs, 2,330–3,095 DS events (FDR < 0.05) were found in 928–1,416 DSGs between seasonal forms at each developmental stage, of which more than half contained MXE or SE sites (supplementary tables S5 and S6, Supplementary Material online). The majority of DSGs do not appear to be DEGs at each developmental stage (Wr60 stage, fig. 2*C*; other stages, supplementary fig. S1, Supplementary Material online), and vice versa.

To elucidate the function of DEGs and DSGs, we performed a functional enrichment analysis. Both the Kyoto Encyclopedia of Genes and Genomes (KEGG) and Gene Ontology (GO) enrichment analyses suggested that DEGs and DSGs between seasonal forms were enriched for distinct pathways and functions at each developmental stage (supplementary fig. S2, Supplementary Material online). During Wr60, the temperature-sensitive stage when eyespot size plasticity is primarily determined (Monteiro et al. 2015), a GO term ecdysteroid metabolic process (*P*adj < 0.0001), among others, was significantly enriched for genes up-regulated in the WS form (*P*adj < 0.05, log2FC > 1) (supplementary fig. S2*A*, Supplementary Material online), whereas a different set of GO terms related to transcriptional regulations, such as regulation of alternative mRNA splicing via spliceosome (*P*adj = 0.0012), and DNA-binding transcription factor activity (*P*adj = 0.0022), were significantly enriched for DSGs between seasonal forms at the same Wr60 stage (FDR < 0.05, |ΔPSI| > 0.1) (supplementary fig. S2*C*, Supplementary Material online). Similarly, the KEGG pathway insect hormone biosynthesis (*P*adj = 0.0186) was significantly enriched for genes up-regulated in the WS form (*P*adj < 0.05, log2FC > 1) (supplementary fig. S2*B*, Supplementary Material online), whereas different KEGG pathways such as Hippo signaling pathway-fly (*P*adj = 0.085) and MAPK signaling pathway-fly (*P*adj = 0.076) were enriched for DSGs between seasonal forms during Wr60 (FDR < 0.05, |ΔPSI| > 0.1) (supplementary fig. S2*D*, Supplementary Material online). These results suggest that DEGs and DSGs are different sets of genes with distinct functions in the seasonal forms.

### Some Eyespot-Related Genes are Also DE or DS Between Seasonal Forms

**Fig. 3.**
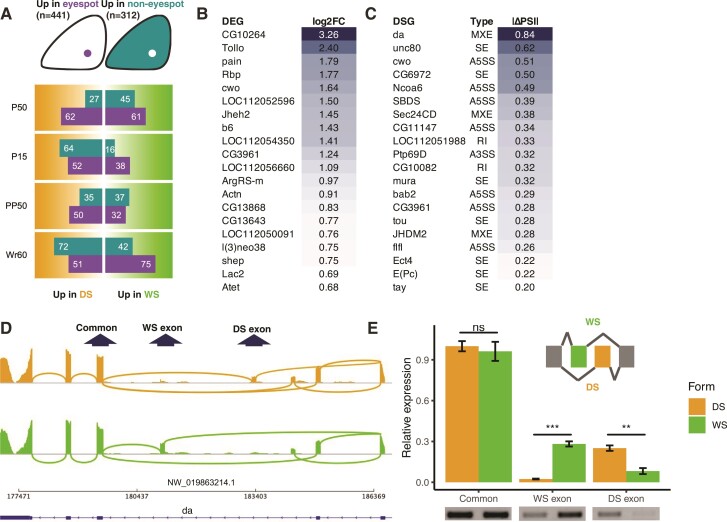
DEGs and DSGs associated with eyespots, and the splicing pattern of an eyespot gene *daughterless* in the seasonal forms. (*A*) Number of genes differentially expressed between eyespot and non-eyespot tissue that are also differentially expressed between seasonal forms at each developmental timepoint. The eyespot-associated genes were obtained from two previous studies identifying (forewing Cu1) eyespot-associated genes in early pupal forewings (Özsu and Monteiro 2017; Murugesan et al. 2022). (*B*) and (*C*) show the top 20 differentially expressed (largest fold-changes between WS vs. DS forms), and differentially spliced (largest absolute values of inclusion level differences between seasonal forms) eyespot up-regulated genes, respectively, between seasonal forms during Wr60. (*D*) Major splicing junctions (read counts > 30) were shown for the top DSG, *daughterless* (*da*), suggesting that *da* has two seasonal-form-specific exons. (*E*) qPCR and regular PCR followed by gel electrophoresis was used to quantify the relative expression of the WS exon, DS exon, and a downstream common exon in the seasonal forms during Wr60. *Rps18* was used as a housekeeping gene in the qPCR analysis. Error bar indicates standard error of the mean. Statistical significance was assessed via Student’s *t*-test. ns, not significant; *, *P* < 0.05; **, *P* < 0.01; ***, *P* < 0.001.

We were particularly interested in examining the expression level and splicing patterns of genes that are differentially expressed in eyespots compared with noneyespot wing tissues. In two previous studies, a total of 753 genes were identified as showing differential expression between eyespot and noneyespot tissues in forewings of WS forms during the early pupal stage (3 h after pupation), when microdissections of wing tissue can be performed (Özsu and Monteiro 2017; Murugesan et al. 2022). From these, 441 were up-regulated and 312 were down-regulated in eyespots compared with non-eyespot tissue (fig. 3*A*). We used this list of 753 DE eyespot genes in all the subsequent eyespot-related analysis, as any DE or DS patterns found between seasonal forms for these genes might underly eyespot size plasticity.

We discovered that some of the DE eyespot genes were also DE between seasonal forms at each developmental stage (fig. 3*A*, supplementary table S7, Supplementary Material online). During Wr60, when eyespot size is primarily determined (Monteiro et al. 2015), there was a higher number of eyespot up-regulated genes in the WS form (*n* = 75) than in the DS form (*n* = 51), and a higher number of eyespot down-regulated genes in the DS form (*n* = 72) than in the WS form (*n* = 42). Genes up-regulated in the eyespot could be essential for eyespot formation and differentiation, whereas those down-regulated in the eyespot could be eyespot repressors. Such bias was not observed in other stages (fig. 3*A*). Therefore, a larger number of eyespot up-regulated genes in the WS forms, with larger eyespots, and a larger number of eyespot down-regulated genes in the DS form with smaller eyespots, during the critical temperature-sensitive stage Wr60, is compelling evidence that these eyespot-associated genes might underly eyespot size plasticity in the seasonal forms (fig. 3*A*).

Many DE eyespot genes were also DS between seasonal forms at each developmental stage (supplementary table S7, Supplementary Material online), indicating that alternative splicing might also contribute to the regulation of eyespot size plasticity. We did not pursue a similar quantitative analysis of the DSGs observed because there is no clear biological meaning associated with variation in the frequency of splice forms, short of using functional tools.

We then examined to what extent eyespot DE and DS genes overlap between seasonal forms at each developmental stage. Among the whole set of eyespot genes that are either DE or DS between seasonal forms at each developmental stage, no more than 15% are both DE and DS (supplementary table S7, Supplementary Material online), suggesting that, as for the whole gene set, most eyespot genes that are DE, belong to a different set relative to those that are DS, between seasonal forms.

To examine the potential roles of some of these eyespot DEGs or DSGs, we highlighted the top 20 eyespot up-regulated genes that were also up-regulated in the WS form (fig. 3*B*), and the top 20 eyespot up-regulated genes that were also DS between seasonal forms (fig. 3*C*) during Wr60, the temperature-sensitive stage when eyespot plasticity is primarily determined (Monteiro et al. 2015).

### Eyespot Gene *Daughterless* is not a DEG but a DSG in the Seasonal Forms

The top eyespot up-regulated gene that was also DS between seasonal forms is *daughterless* (*da*) (|ΔPSI| = 0.84, FDR < 0.05, fig. 3*C*), with a mutual exclusive splicing pattern. Major RNA-seq read junctions (read counts > 30) linking adjacent exons of *da* are visualized in figure 3*D*. There are two newly discovered *da* exons that are not previously annotated in the NCBI genome (GCF_900239965.1), one primarily expressed in the DS form (DS exon), the other in the WS form (WS exon) (fig. 3*D*). Both quantitative polymerase chain reaction (qPCR) and regular PCR were performed to quantify the relative expression of the WS exon, DS exon, and a downstream common exon of *da* in the seasonal forms during Wr60 (fig. 3*D*). qPCR results showed that the common exon was expressed at an equivalent level in the seasonal forms (*P* = 0.576), supported by the RNA-seq data that *da* is not a DEG (*P*adj > 0.05). The WS exon was expressed, however, at significantly higher levels in the WS form (*P* < 0.001), and the DS exon at higher levels in the DS form (*P* < 0.01) (fig. 3*E*). This result was supported by a regular PCR gel electrophoresis, where bands of equivalent intensity were observed for the common exon, and stronger bands were observed for the seasonal form-specific exons in the corresponding seasonal forms (fig. 3*E*). The results suggest that *da* is not DE between seasonal forms but exhibits a mutually exclusive splicing pattern in the seasonal forms during Wr60, in response to rearing temperatures.

### Some Primary 20E Response Factors are Also Eyespot-Related Genes, Many of Which are DS But Not DE Between Seasonal Forms

To understand how 20E response factors react to 20E pulses, we examined the dynamic expression patterns of seven well-studied primary 20E response genes, *EcR*, *Ultraspiracle* (*Usp*), *Broad complex* (*Br-C*), *Ecdysone-induced factors E74*, *E75*, *E93*, and *Fushi tarazu transcription factor 1* (*Ftz-f1*), in the seasonal forms throughout wing development (Song and Zhou 2020) (supplementary fig. S3*A*, Supplementary Material online). We observed that multiple primary 20E response factors *EcR*, *Usp*, *E74*, and *Ftz-f1*, showed significant up-regulations during the transition from Wr60 to PP50, spanning the larval 20E pulse, in both seasonal forms. In contrast, down-regulations were observed for most of these factors during the transition from P15 to P50, spanning the pupal 20E pulse (supplementary fig. S3*A*, Supplementary Material online). This suggest that primary 20E response factors could be short lived and their rapid response to 20E might only be captured across the shorter interval that flanks the larval 20E pulse, but not across the longer interval that flanks the pupal pulse (1 day and 2.5 days, respectively for WS forms). These primary 20E response factors might have dropped to a basal level, or below, before the time 20E also drops to a basal level during P50.

We then checked whether some of these primary 20E response factors were also DE eyespot genes or previously shown to exhibit eyespot-related expression patterns. We found that four genes, *EcR* (Mateus et al. 2014; Monteiro et al. 2015), *Usp* (Bhardwaj 2018), *Br-C* (Mateus et al. 2014), and *Ftz-f1* (in the DE eyespot gene list), were DE (up-regulated) in eyespots. We then examined whether they were DE or DS between seasonal forms, before (Wr60) or after (PP50) the larval 20E pulse, when hindwing eyespot size is primarily determined (Monteiro et al. 2015). Only *Br-C* showed marginally higher expression in DS forms compared with WS forms during PP50 (log2FC = −0.44, *P*adj < 0.05) (supplementary fig. S3*A*, Supplementary Material online). However, *EcR*, *Usp*, and *Br-C* were all DS between seasonal forms both at Wr60 and at PP50 with very few differences across the two stages. This suggests that these DS patterns might be independent of 20E signaling, but a response to temperature instead (supplementary fig. S3*B*, Supplementary Material online). The DS of *Ftz-F1*, however, might be triggered by the 20E pulse because the DS pattern only appeared at PP50, but not at Wr60 (supplementary fig. S3*B*, Supplementary Material online). These results suggest that these four primary 20E response genes are highly expressed in eyespots of both seasonal forms, but their expression level is mostly comparable across seasonal forms. These genes, however, are DS mostly in response to temperature, rather than 20E, between seasonal forms.

### Developmental Transitions Cued by 20E Pulses have a Larger Impact in Remodeling miRNA Expression than Rearing Temperatures

Because sRNAs play an important role in the post-transcriptional regulation of gene expression, we performed sRNA-seq on the same samples used for RNA-seq to elucidate the expression patterns of sRNAs in the seasonal forms of *B. anynana*.

**Fig. 4.**
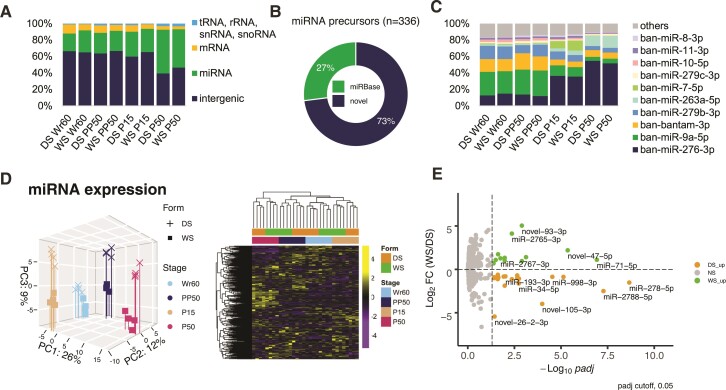
Small RNA compositions and expression of miRNAs in the seasonal forms during wing development. (*A*) The composition of the small RNA population in *B. anynana* developing wings. (*B*) The proportion of novel and conserved miRNAs annotated in *Bicyclus anynana.* (*C*) The top 10 highly expressed miRNAs in the seasonal forms across wing development. (*D*) PCA and hierarchical clustering heatmap showing the global miRNA expression similarity in the seasonal forms throughout wing development using all miRNAs. (*E*) The volcano plot highlights DEmiRs between seasonal forms during Wr60.

After adaptor trimming, the raw sRNA-seq reads exhibited a bimodal distribution, peaking at 22nt and 28nt, which are the typical lengths of miRNAs and piwi-interacting RNAs (piRNA), respectively (supplementary fig. S4, Supplementary Material online). Major types of sRNAs were already annotated in the *B. anynana* v1.2 genome (GCF_900239965.1, see Materials and Methods for more detail). Since miRNAs had never been annotated in *B. anynana* before, we annotated the first set of miRNAs in *B. anynana*, and then determined how the composition of sRNAs varied across developmental stages and seasonal forms. The results suggest that the sRNA population in *B. anynana* is mainly composed of miRNAs and intergenic sRNAs, generally considered piRNAs (Jehn et al. 2018), and the composition of sRNAs is stable across developmental stages and seasonal forms (fig. 4*A*).

We then focused on miRNAs and investigated to what extent they were previously annotated in other species. Within the set of 336 precursor miRNAs that were annotated in *B. anynana*, 91 (27%) are conserved, with orthologous miRNAs found in other species registered in miRBase (Griffiths-Jones et al. 2006), whereas the rest are novel to *B. anynana* (fig. 4*B*). Mature sequences of all miRNAs and the novel predicted miRNAs showed 5′U preference, a feature of genuine miRNAs (Lau et al. 2001) (supplementary fig. S5, Supplementary Material online).

We then examined how miRNAs varied between seasonal forms and developmental stages. In each sequencing library, the total miRNA population is mainly comprised of several highly expressed miRNAs, and the composition of the highly expressed miRNAs varies primarily across developmental stages (fig. 4*C*). In the PCA of miRNA expression, PC1 and PC2 (cumulatively explaining 38% of total variance) separate the four developmental stages, whereas PC3 (9% of total variance) separates the seasonal forms (fig. 4*D*, left). As previously observed for the gene expression and splicing patterns, miRNAs are primarily clustered by developmental stage, then by seasonal form (fig. 4*D*, right). Also, Wr60 and P15, the two stages before the larval and pupal 20E pulses, cluster more closely together than PP50 and P50, the two stages after the 20E pulses (fig. 4*D*, right). This suggest that, as for gene expression and alternative splicing, developmental transitions that coincide with 20E pulses, also have a larger impact in remodeling miRNA expression than rearing temperatures. The differentially expressed miRNAs (DEmiRs) between seasonal forms, at each developmental stage, were summarized in supplementary tables S8 and S9, Supplementary Material online. DEmiRs between seasonal forms during Wr60 are highlighted in figure 4*E*.

### DEG–DEmiR Regulatory Network Reveals Extended miRNA–Gene Regulation Between Seasonal Forms

**Fig. 5.**
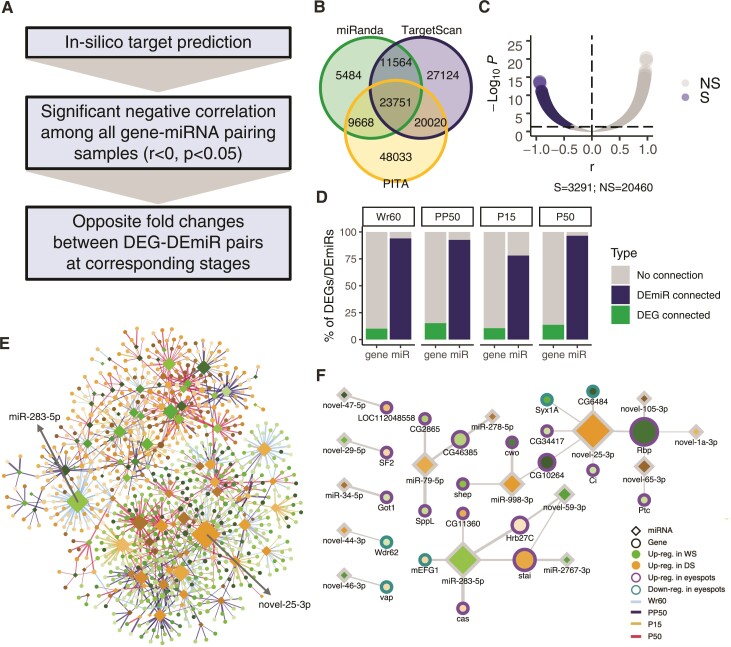
The construction of a DEG–DEmiR regulatory network between seasonal forms. (*A*) A pipeline was used to predict high-confidence regulatory interactions between DEGs and DEmiRs in the seasonal forms. (*B*) First, three in silico tools, TargetScan, miRanda, and PITA, were used to search binding sites of pooled DEmiRs in the 3′UTRs of pooled DEGs between seasonal forms from all developmental timepoints examined. (*C*) The predicted targeting pairs were then filtered for a negative correlation (Pearson correlation *r* < 0, *P* < 0.05) between expression levels of paired genes and miRNAs across all samples, generating 3,291 validated pairs. S, significant; NS, not significant. (*D*) Finally, the validated DEG and DEmiRs should show opposite directions of fold-changes between seasonal forms at the corresponding stages, generating a final list of DEG–DEmiR interactions between seasonal forms. Bar plot shows the proportion of DEGs and DEmiRs involved in the DEG–DEmiR regulatory network split by developmental stage. (*E*) The complete network. The top two hub miRNAs are labeled. (*F*) A proportion of the network showing eyespot-associated genes predicted to be under direct control of miRNAs during Wr60.

MiRNAs elicit their inhibitory effect on gene expression by binding 3′UTRs of target mRNAs to mediate mRNA decay. To investigate whether some of the DEGs are direct targets of DEmiRs, a pipeline was used to construct putative gene–miRNA regulatory networks (fig. 5*A*). First, all DEGs and DEmiRs between seasonal forms from each stage were pooled. DEG–DEmiR targeting pairs were predicted by searching for miRNA binding sites of DEmiRs in the 3′UTRs of DEGs, using three in silico prediction tools: miRanda (Enright et al. 2003), TargetScan (Lewis et al. 2003), and PITA (Kertesz et al. 2007). In total, 23,751 targeting pairs were predicted by all three tools (fig. 5*B*). Since a higher expression level of a miRNA should correspond to a lower expression level of its direct targets, the predicted targeting pairs were further filtered by selecting those with significant negative correlations (Pearson correlation *r* < 0, *P* < 0.05) between the expression levels of the paired genes and miRNAs across all sequencing samples, generating 3,291 validated pairs (fig. 5*C*). Finally, opposite directions of fold-changes between validated DEG–DEmiR pairs would be expected when DEmiRs regulate DEGs between seasonal forms in the corresponding stages. The analysis showed that ∼10% of DEGs at each stage are putatively regulated by over 70% DEmiRs, which suggest an essential role of miRNAs in regulating DEGs in *B. anynana* seasonal forms (fig. 5*D*).

The predicted DEG–DEmiR regulatory network consists of two clusters, one with miRNAs up-regulated in the WS form with their direct targets down-regulated in the WS form, and the other showing the opposite pattern (fig. 5*E*). Some miRNAs are DE between seasonal forms only at a certain developmental stage, and regulate target DEGs in that stage, whereas others are DE across multiple stages and target the same or different sets of genes across multiple stages. Two miRNAs with the highest degree of connectivity are miR-283-5p, up-regulated in the WS form during Wr60, and novel-25-3p, up-regulated in DS forms across all four stages. All the DEmiRs that were predicted to regulate DEGs between seasonal forms are listed in supplementary table S9, Supplementary Material online.

To investigate the subset of genes and corresponding miRNA that might be regulating eyespot size plasticity, we plotted a DEG–DEmiR regulatory network involving eyespot-associated genes only during Wr60 (fig. 5*F*). This network involves 22 genes associated with eyespots, of which 17 are up-regulated in eyespots. These results suggest that multiple eyespot genes, DE between seasonal forms, are potentially under direct control of miRNAs that are also DE. This suggests a potential miRNA–gene regulatory mechanism underlying eyespot size plasticity in *B. anynana*.

## Discussion

### Multiple Transcriptomic Patterns Coordinately Respond to Developmental Transitions Cued by 20E Pulses and Rearing Temperatures

In this study, we examined how developmental transitions, cued by 20E pulses, and rearing temperatures shaped multiple transcriptomic patterns, including gene expression, alternative splicing, and miRNA-mediated gene silencing, in *B. anynana* hindwing tissue. We observed a stronger genome-wide response to developmental transitions than to rearing temperatures across all the transcriptomic patterns assessed (figs. 2*A*, *B* and 4*D*). Moreover, all these transcriptomic patterns are more similar pre-20E pulses, during Wr60 and P15, than post-20E pulses, during PP50 and P50, suggesting that 20E could be an important regulator of multiple transcriptomic patterns in a stage-specific way (figs. 2*A*, *B* and 4*D*).

Transcriptomic profiles are closely intertwined with chromatin accessibility during insect metamorphosis (Mukherjee et al. 2012), and chromatin remodeling is potentially mediated by 20E signaling. We observed that multiple primary ecdysone response factors, *EcR*, *Usp*, *E74*, and *Ftz-f1*, showed significant up-regulations during the transition from Wr60 to PP50 (supplementary fig. S3, Supplementary Material online). Three of these factors (*EcR*, *Usp*, and *Ftz-f1*) were also up-regulated in another butterfly, *Junonia coenia*, during the same larval–prepupal transition, concurrently with a strong motif enrichment in open chromatin regions for the recognition sequences of these factors (van der Burg et al. 2019). This suggests that chromatin remodeling, potentially mediated by ecdysone response factors, might bring about massive transcriptomic changes post-20E pulses. It also explains why transcriptomic profiles are most similar pre-20E pulses, during Wr60 and P15, when changes in the chromatin accessibility have not yet taken place.

**Fig. 6.**
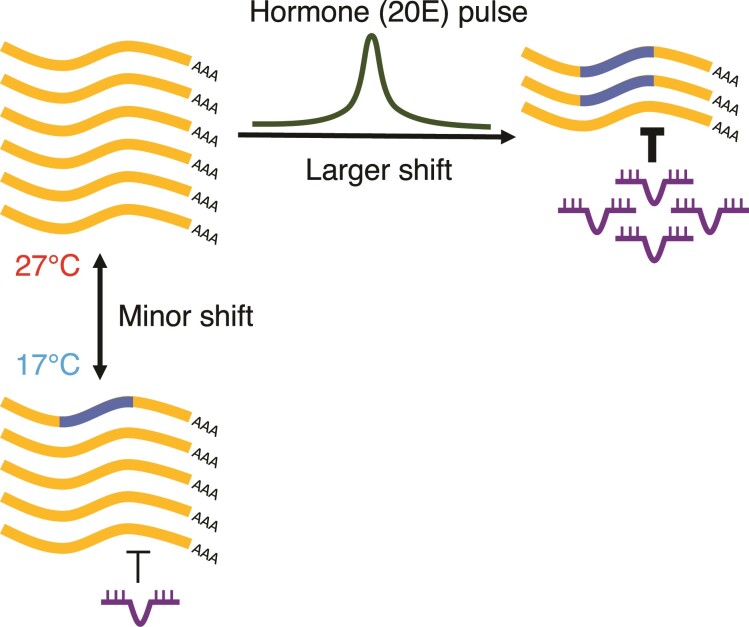
Developmental transitions cued by 20E pulses, and rearing temperatures coordinately remodel the transcriptomic landscape. Transcriptomic patterns, including gene expression, alternative splicing, and miRNA-mediated gene silencing, are coordinately remodeled by both developmental transitions, cued by hormone (20E) pulses, and rearing temperatures. 20E pulses appear to induce a substantially larger shift in transcriptomic patterns than rearing temperatures per se.

Environmental cues such as temperature, on the other hand, can also remodel chromatin status, and impact trait plasticity in insects (Gibert et al. 2007). Our results suggest that the chromatin remodeling induced by both developmental transitions and temperatures might coordinately impact both gene expression and various post-transcriptional features, including alternative splicing and miRNA expression, with a larger impact attributed to developmental transitions, cued by hormone pulses (fig. 6). The extent that each of these transcriptomic differences is involved in the process of producing plastic wing pattern morphologies, remains to be investigated.

### Post-transcriptional Regulations are Understudied Mechanisms Underlying Developmental Plasticity

We showed that 36% of the total *B. anynana* transcriptome can be alternatively spliced, which is a comparable proportion to that observed in some invertebrates such as *Drosophila melanogaster*, *Nematostella vectensis*, and pea aphids (Chen et al. 2014). We also observed a higher proportion of splicing events being MXE and SE, the two types that are more likely to produce functional proteins, as they are more likely to retain an open reading frame (Weatheritt et al. 2016; Grantham and Brisson 2018). Moreover, these two types also constitute over half of the DS events observed between seasonal forms. These data suggest that diverse proteins generated via alternative splicing may play a role in regulating phenotypic plasticity in *B. anynana*.

We also showed that DEGs and DSGs are distinct sets of genes between seasonal forms, which were also observed in the polyphenic pea aphids (Grantham and Brisson 2018). Differential gene expression produces the same gene products with different abundance, whereas alternative splicing produces functionally distinct protein products with similar abundance. Our results indicate that distinct sets of genes undergo these two types of regulations, and each set might mediate divergent functional changes in the seasonal forms. Taken together, differential gene expression and alternative splicing appear to work independently, rather than synergically, in response to environmental cues that underly plastic traits.

In this study, we also annotated the first set of miRNAs in *B. anynana*. Most of the miRNAs annotated are novel, supported by evidence that there exists a burst of miRNA innovations in the early radiation of lepidoptera (Quah et al. 2015; Ma et al. 2021). We discovered that around 10% of the total DEGs are putatively direct targets of DEmiRs between seasonal forms, suggesting an essential role of miRNAs in post-transcriptional gene regulation in the seasonal forms. The proportion of DEGs regulated by DEmiRs may be underestimated because of the stringent filtering steps adopted to find high-confidence gene–miRNA interactions.

### DE or DS Eyespot Genes, and DEmiRs Regulating Eyespot genes, are Putative Eyespot Size Regulators

We found many eyespot up-regulated genes that are also up-regulated in the WS form during Wr60, when eyespot size plasticity is primarily determined (fig. 3*B*) (Monteiro et al. 2015). Top candidates exhibiting the largest expression fold-changes between the seasonal forms, highlight some of the genes that could potentially be involved in eyespot size plasticity. *Tollo* encodes a Toll-like receptor protein involved in immune responses in *Drosophila* (Akhouayri et al. 2011). A recent study identified the Toll signaling pathway as potentially involved in eyespot formation in *B. anynana* (Özsu and Monteiro 2017). Thus, higher Tollo expression in the WS form could potentially lead to larger eyespots. *Painless* (*pain*) encodes a cation channel protein, that is, sensitive to high temperature in *Drosophila* (Sokabe and Tominaga 2009). Eyespot expression of *pain* and its higher expression in the WS form suggest that this sensor gene could possibly be wired to the eyespot gene regulatory network to make WS eyespots hypersensitive to elevated temperature, thereby regulating eyespot size in direct response to rearing temperatures. Juvenile hormone (JH) expoxide hydrolase 2 (Jheh2) catalyzes JH hydrolysis (Share and Roe 1988). Since JH signaling and 20E signaling are antagonistic, higher *Jheh2* expression in the eyespots in the WS form could potentially reduce local JH levels in the eyespot centers and facilitate higher 20E signaling in the WS form during Wr60 to produce larger eyespots.

Some of the primary 20E response genes that also appeared as eyespot up-regulated genes, such as *EcR*, *Usp*, and *Ftz-F1*, were not DE between seasonal forms at both Wr60 and PP50. This suggests that temperature leads to higher 20E titers in WS forms, but perhaps both the DS form and the WS form titers are sufficient to drive the expression of primary 20E response genes in similar amounts. It is then the 20E titer difference, per se, that might create the 20E signaling asymmetry affecting downstream targets, and eyespot size plasticity in the two seasonal forms, as previously suggested (Monteiro et al. 2015).

We also noticed that a gene involved in the final step of 20E biogenesis, *shade* (*shd*) (Petryk et al. 2003), and another gene encoding a channel protein that facilitates the importation of 20E from hemolymph into cells, *Ecdysone Importer* (*EcI*) (Okamoto et al. 2018), are up-regulated in the WS form during Wr60 (supplementary table S4, Supplementary Material online). Although these genes do not appear to be eyespot genes, they may induce an overall higher 20E production and a higher 20E sensitivity across WS wings, promoting the formation of larger eyespots in the WS form.

For eyespot genes that are DSGs (fig. 3*C*), we highlighted *da*, a gene encoding a class I basic helix-loop-helix (bHLH) protein with diverse roles in insect development, including sex determination, neural differentiation, and oogenesis (Cline 1978; Caudy et al. 1988; Cummings and Cronmiller 1994). In this study, *da* was predicted to have a mutual exclusive splicing pattern between seasonal forms, during Wr60, in response to rearing temperatures. Both qPCR and regular PCR followed by gel electrophoresis successfully validated this pattern. In *Drosophila*, Da cooperates with two other bHLH proteins, Achaete and Scute, to control the development of sensory bristles (Caudy et al. 1988; Jafar-Nejad et al. 2006). Since insect sensory bristles are homologous to butterfly wing scales (Galant et al. 1998; Connahs et al. 2019), we propose that Da also plays a role in butterfly scale development.

Apart from *da*, multiple eyespot up-regulated genes that are primary 20E response factors, such as *EcR*, *Usp*, *Br-C*, and *Ftz-F1*, were also DS between seasonal forms during Wr60 and/or PP50, although most of them were not DE. The exact roles of these alternatively spliced proteins in each seasonal form, however, need to be further investigated using functional tools.

The most significant DEmiRs between seasonal forms, during Wr60, were miR-278-5p and miR-2788-5p, both up-regulated in the DS form (fig. 4*E*). The counterpart strand of miR-278-5p, miR-278-3p, is known to repress JH signaling by targeting a JH early response gene *Krüppel-homolog 1* (*Kr-h1*) (Song et al. 2018). Although it is unknown whether miR-278-5p also regulates JH signaling in *B. anynana*, the DEG–DEmiR regulatory network suggests that miR-278-5p directly represses an eyespot gene *CG46385* in the DS form (fig. 5*F*). CG46385 was predicted to be an adenylyltransferase with yet unknown roles in eyespot development. *Mir-2788* is one of the two miRNAs located within the *HmYb* region, a genomic locus associated with the hindwing yellow bar pattern in *Heliconius melpomene* butterflies (Surridge et al. 2011). Future functional studies should investigate the potential role of *mir-2788* in the development of butterfly wing patterns, in both polyphenic and polymorphic species. Multiple miRNAs were predicted to directly target eyespot genes that were DE between seasonal forms. Some of these miRNAs are predicted to target multiple eyespots genes, and individual eyespot genes were also predicted targets of multiple miRNAs. It is possible that some of these miRNAs might underly eyespot size plasticity in the *B. anynana* seasonal forms.

### Limitations and Future Directions

Some limitations of our interpretations of the genetic basis of hindwing eyespot size plasticity include cross-referencing a list of DEGs between forewing Cu1 eyespots and adjacent noneyespot tissue from the early pupal forewings, at 3 h postpupation, in our current study that investigates transcriptomic patterns in both larval as well as pupal hindwings. By doing this we assumed that forewing and hindwing eyespots, at different developmental stages, share the same set of eyespot genes with similar functions, which is not necessarily true. For instance, a homeobox gene, *Ultrabithorax* (*Ubx*), is only expressed in hindwings but not in forewings, and yet is essential for hindwing eyespot development (Matsuoka and Monteiro 2021). Another homeobox gene, *Antennapedia* (*Antp*), has a different function in forewing and hindwing eyespots; when *Antp* is disrupted, eyespots disappear from forewings but only become smaller and without a white center in hindwings (Matsuoka and Monteiro 2021). Therefore, this cross-referencing might have biased our interpretations. Moreover, there is limited resolution to infer changes in omics patterns in the relatively small eyespot regions when whole wing RNA-seq and sRNA-seq datasets are used, as both gene and miRNA expression are highly cell/tissue-specific in insects (Aboobaker et al. 2005; Li et al. 2022). Lastly, many of the eyespot-related patterns described might not directly relate to eyespot size plasticity or other morphological differences observed across seasonal forms, but rather to a general physiological response of the whole wing tissue to temperature (Daniels et al. 2014).

Future functional studies are needed to validate the functions of the candidate genes/miRNAs proposed for regulating hindwing eyespot plasticity. Moreover, future studies should examine the omics patterns specifically in the eyespot region across seasonal forms, which will involve more sophisticated dissections of eyespot cells from larval wings. The omics patterns of color- or pigmentation-related genes or miRNAs between seasonal forms, should be investigated especially during P15 and P50, the two pupal stages when butterfly wing color plasticity is determined (Daniels et al. 2014). This could provide more insights on how the background wing color differences are regulated in the seasonal forms of *B. anynana* and other species with seasonally plastic wing colors. Overall, we generated a comprehensive transcriptomic atlas in a model system of seasonal plasticity at four key points in development, which will aid in deciphering the molecular mechanisms underlying phenotypic plasticity.

## Materials and Methods

### Insect Husbandry

The wild-type lab population of *B. anynana* was reared in two climate rooms at 27 °C and 17 °C, leading to the development of WS and DS forms, respectively. Both climate rooms have a 12:12 light: night cycle with 60% relative humidity. Larvae were fed young corn leaves and adult butterflies were fed mashed banana on moist cotton.

### Developmental Staging of the Seasonal Forms

A precise developmental staging method was adopted to sample wings from equivalent developmental timepoints across the two seasonal forms. Fifth instar female larvae were reared in individual transparent containers with corn leaves, and imaged every 30 min using the time-lapse function of an Olympus Tough TG-5 camera. The initiation of the Wr stage happened when the larva stopped feeding, leaving the food, and climbing up to the cap or the inner wall of the container. The initiation of the PP stage happened when the larva began hanging upside down and became J-shaped. The initiation of the P and A stages were marked by the pupal and adult eclosion, respectively. The transition start times and durations of the Wr, PP, P, and A stages (no durations recorded for the A stage) were recorded for both seasonal forms, involving 20–41 individuals in each measurement.

Since the transition start times and durations were highly consistent with photoperiods in the WS forms (fig. 1*B* and *C*), WS individuals were dissected exactly at the expected dissection time of the day according to the staging h/days (fig. 1*E*). However, the transition start times (except that for the A stage) were highly dispersed and unpredictable in the DS forms (fig. 1*B*), but the durations were quite constant, although prolonged compared with the WS form (fig. 1*C*). Therefore, DS individuals were imaged individually to precisely determine their transition start times and were staged individually (fig. 1*E*). As a result, the expected dissection time could vary from individual to individual for the DS individuals. Since photoperiod itself might also affect hormone titers and transcriptomic patterns in insects (Schiesari et al. 2011), we dissected DS individuals only if their expected dissection times were comparable to the expected dissection times of the WS individuals sampled from the same stage, to minimize a potential circadian effect difference between seasonal forms.

### Sample Preparation and Sequencing

WS and DS hindwings were collected from four developmental time points, 60% Wr stage (Wr60), 50% PP stage (PP50), 15% P (P15), and 50% P stage (P50). Each condition consists of four biological replicates, with four hindwings (both left wings and right wings) pooled from two individuals in each replicate. All wings were sampled from females. Fresh tissues were kept in RNAlater solution at 4 °C overnight and stored at −80 °C.

Total RNAs were extracted using mirVana miRNA Isolation Kit, following the total RNA isolation procedure. The total RNA extracted was equally divided into two separate tubes, one for RNA-seq and the other for sRNA-seq. All the total RNA samples were checked for quantity and integrity by Nanodrop, gel electrophoresis, and Agilent 2100. For RNA-seq, mRNA libraries were constructed using the NEBNext Ultra Directional RNA Library Prep Kit. Library quality was assessed by Qubit 2.0, Agilent 2100, and q-PCR. Over 30 million 150 bp paired-end reads were sequenced from each biological replicate using NovaSeq 6000. For sRNAs-seq, sRNA libraries were constructed using the NEBNext Multiplex Small RNA Library Prep Set. Library quality was assessed by Qubit 2.0, Agilent 2100, and q-PCR. Over 20 million 50 bp single-end reads were sequenced for each biological replicate using NovaSeq 6000. The quality control for total RNA samples, library construction, and Illumina sequencing was carried out by NovogeneAIT, Singapore.

### Differential Gene Expression Analysis

Trimmomatic 0.39 (Bolger et al. 2014) was used to trim adaptors from the raw sequencing data to generate clean reads (options: PE ILLUMINACLIP:TruSeq3-PE.fa:2:30:10:8:true MAXINFO:40:0.2). The MAXINFO option was used to favor longer reads over read correctness since the quality of the raw reads was high. Quality control checks were performed using FastQC 0.11.5 before and after adaptor trimming. For differential gene expression analysis, gene models were obtained from the NCBI *B. anynana* v1.2 genome (Nowell et al. 2017) (GCF_900239965.1). Gene models were downloaded from: https://ftp.ncbi.nlm.nih.gov/genomes/all/annotation_releases/110368/100/. Clean reads were used to quantify all the annotated transcripts using Salmon 1.2.1 (Patro et al. 2017) with the quasi-mapping mode (options: –validateMappings –seqBias –gcBias). Trimming and Salmon mapping statistics were summarized in supplementary table S10, Supplementary Material online. Raw transcript counts were imported in R studio and converted to gene-level counts using the R package tximport (Soneson et al. 2015). The raw gene counts were normalized, and DE analysis was performed using DESeq2 (Love et al. 2014) in R studio. One of the RNA-seq libraries, DS2_P15, appeared as an obvious outlier, thus was excluded in all subsequent analysis (supplementary fig. S6, Supplementary Material online).

### Functional Annotation and Enrichment Analysis

A local blastx was performed to blast all the CDS regions of the annotated genes in the NCBI *B. anynana* v1.2 genome (GCF_900239965.1) against a nonredundant (nr) protein database using diamond 0.9.30 (Buchfink et al. 2015) (options: -e 1e-5 -f 5 -k 20). The annotation result was imported in Omicsbox, and blast2GO (Conesa et al. 2005) was run to get the GO (Ashburner et al. 2000) annotations. Meanwhile, InterProScan (Jones et al. 2014) and EggNOG mapper (Huerta-Cepas et al. 2019) were also run for all the CDS regions using Omicsbox. The resulting GO terms from blast2GO, InterProScan, and EggNOG were merged using Omicsbox. The KEGG (Kanehisa and Goto 2000) annotation was obtained directly from the EggNOG mapper results. GO and KEGG pathway enrichment analysis was performed using the R package clusterprofiler (Yu et al. 2012).

### Alternative Splicing Analysis

Clean RNA-seq reads were aligned to the *B. anynana* v1.2 genome using HISAT2 2.1.0 (Kim et al. 2015). HISAT2 alignment statistics were summarized in supplementary table S10, Supplementary Material online. rMATS turbo v4.1.0 (Shen et al. 2014) was used to detect alternative splicing events. rMATS reports six types of alternative splicing events, including SE, A5SS, A3SS, MXE, and RI.

First, alternative splicing events were assessed in each seasonal form and developmental stage separately (options: -t single –readLength 150 –variable-read-length –novelSS). By specifying –novelSS, both annotated splice junctions in the *B. anynana* v1.2 genome and novel junctions were assessed. A sum of 20 counts was required for both inclusion and skipping junctions to support a splice site. To filter out splicing forms of extremely low abundance, which are potentially due to splicing mistakes, confidential splice sites were required to have a mean percentage spliced in (PSI) level (reported as inclusion level by rMATS) over 0.1 and <0.9, as suggested by multiple studies (Wang et al. 2008; Shapiro et al. 2011; He et al. 2015; Grantham and Brisson 2018).

rMATS was also used to assess differential splicing patterns between seasonal forms. Pair-wise comparisons between seasonal forms were made at each developmental stage. Although a sum of 20 counts of both inclusion and skipping junctions was still required to support a splice site, the PSI filter was not applied since there could be spliced forms only existing in one seasonal form but not the other, in this case, with a PSI level of 0 or 1 in one seasonal form. The inclusion level difference (ΔPSI) between seasonal forms (mean PSI of DS forms—mean PSI of WS forms) was reported by rMATS. A splice site was considered DS when FDR < 0.05. A gene was considered DSG if it has at least one DS site between seasonal forms. If a DSG had multiple DS sites, the DS site with the maximum absolute value of ΔPSI was used to represent the differential splicing level of the gene.

### qPCR and Regular PCR Validation of the Differential Splicing Pattern of *Daughterless*

For qPCR, total RNA was extracted from Wr60 hindwings in both seasonal forms using QIAGEN RNeasy Plus Mini Kit. Total RNA from three individuals was pooled as one biological replicate, five biological replicates were included in the qPCR analysis. Total RNA was reverse transcribed into cDNA using SuperScript II Reverse Transcriptase. Three sets of primers were designed to amplify a ∼85 bp amplicon of the DS exon, WS exon, and a downstream common exon of *da* as shown in figure 3*D*. *RpS18* was used as a housekeeping gene. KAPA SYBR FAST qPCR Master Mix was used for qPCR quantification, involving three technical replicates for each biological replicate. The same sets of primers were used for a regular PCR followed by gel electrophoresis to visualize the bands of the amplicons. The primer sequences and their efficiencies in the qPCR analysis were shown in supplementary table S11, Supplementary Material online. An analysis of the stability of the housekeeping gene *RpS18* was shown in supplementary fig. S7, Supplementary Material online.

### sRNA-seq Data Curation and Annotation

Adaptors were trimmed from the raw sequencing data using Trimmomatic 0.39 (options: SE ILLUMINACLIP:TruSeq3-SE.fa:2:30:10). Quality control checks were performed using FastQC 0.11.5 before and after trimming.

To improve miRNA annotation, rRNAs, tRNAs, snRNAs, and snoRNAs were removed by aligning against the corresponding sequences in the NCBI *B. anynana* v1.2 genome (GCF_900239965.1) using SortMeRNA 2.1b (Kopylova et al. 2012). To remove contaminant sequences, rRNAs were further removed by aligning against all the rRNA libraries provided with the SortMeRNA distribution, which includes Rfam 5s, Rfam 5.8s, and Bacterial, Archaea, and Eukaryotic SILVA rRNA Databases (Griffiths-Jones et al. 2003; Quast et al. 2012). tRNAs were further removed by aligning against Rfam tRNA and GtRNAdb (Griffiths-Jones et al. 2003; Chan and Lowe 2009). Then, clean reads between 17 and 25nt were used to annotate miRNAs as described below. The curation statistics of sRNA-seq data for miRNA annotation were summarized in supplementary table S12, Supplementary Material online.

To annotate the complete sRNA composition in *B. anynana*, adaptor trimmed clean reads were aligned to the *B. anynana* v1.2 genome using STAR 2.7.8a (Dobin et al. 2013) (options: –alignIntronMax 1 –outFilterMultimapNmax 100,000 –outFilterMismatchNmax 3). The predicted miRNA precursors, as well as the annotated rRNA, tRNA, snRNA, snoRNA, and mRNA from the *B. anynana* v1.2 genome (GCF_900239965.1) were used to annotate the sRNA composition using unitas 1.7.7 (Gebert et al. 2017).

### miRNA Annotation and Differential Expression Analysis

MiRNAs were annotated and quantified using miRDeep2 0.1.3 (Friedländer et al. 2012). First, clean sRNA-seq reads between 17 and 25nt were mapped to the genome using mapper.pl (options: -d -e -h -l 16 -m -q). The hairpin-like miRNA precursors were then predicted by the miRDeep2.pl module using all the sequencing libraries with default settings. All mature miRNA sequences of *H. melpomene*, *Bombyx mori*, and *D. melanogaster* from miRBase 22.1 (Griffiths-Jones et al. 2006) were used as closely related species to guide the annotation.

The annotated miRNA precursors were considered true positives if they satisfied the following criteria: significant randfold *P*-value; miRDeep2 score >3. To identify conserved and novel miRNAs, mature miRNAs were blasted against miRBase 22.1, those with a hit were considered conserved miRNAs, whereas those with no hit were considered novel miRNAs. We found identical or highly similar miRNAs derived from different genomic loci. To generate a nonredundant set of mature miRNAs for naming purposes, all mature miRNAs were blasted against themselves to find identical or similar sequences. All conserved miRNAs were manually named under the miRNA nomenclature guidelines (Desvignes et al. 2015), whereas a prefix “novel” was used for novel miRNAs. The complete annotation of miRNAs in *B. anynana* hindwing tissue was summarized in supplementary table S13, Supplementary Material online.

MiRNA expression was quantified using the quantifier.pl module of miRDeep2. Total miRNA counts were normalized, and DE analysis was performed using DESeq2 in R studio.

### miRNA Target Prediction and the Construction of a Gene-miRNA Regulatory Network

A stringent pipeline was designed to select high-confidence miRNA–gene targeting pairs. First, 3′UTR regions were extracted from all the DEGs between seasonal forms and were searched for targeting sites of all the DEmiRs between seasonal forms, using three de novo miRNA target prediction tools, TargetScan (Lewis et al. 2003), miRanda (Enright et al. 2003), and PITA (Kertesz et al. 2007). The resulting miR–gene targeting pairs successfully predicted by all three tools were filtered for a significant negative correlation (Pearson correlation *r* < 0, *P* < 0.05) between expression levels of paired genes and miRNAs across all sequencing samples. Finally, the validated gene–miRNA pairs were considered true if DEGs and DEmiRs showed opposite directions of fold-change between seasonal forms at the corresponding developmental stage. The resulting DEmiR–DEG network was visualized using Cytoscape (Shannon et al. 2003).

## Supplementary Material

Supplementary data are available at *Molecular Biology and Evolution* online.

## Supplementary Material

msac126_Supplementary_Data

## Data Availability

All Illumina reads of RNA-seq and sRNA-seq are available under NCBI BioProject PRJNA844216 (http://www.ncbi.nlm.nih.gov/bioproject/844216).
