# Supplementary material for: A Transcriptomic Atlas Underlying Developmental Plasticity of Seasonal Forms of Bicyclus anynana Butterflies
Source: Mol Biol Evol. 2022 Jun 9;39(6):msac126. doi: 10.1093/molbev/msac126 (PMC9218548; doi:10.1093/molbev/msac126)
Supplement: msac126_Supplementary_Data [file msac126_supplementary_data.zip › Sup.Figures.pdf]

Supplementary Figures.

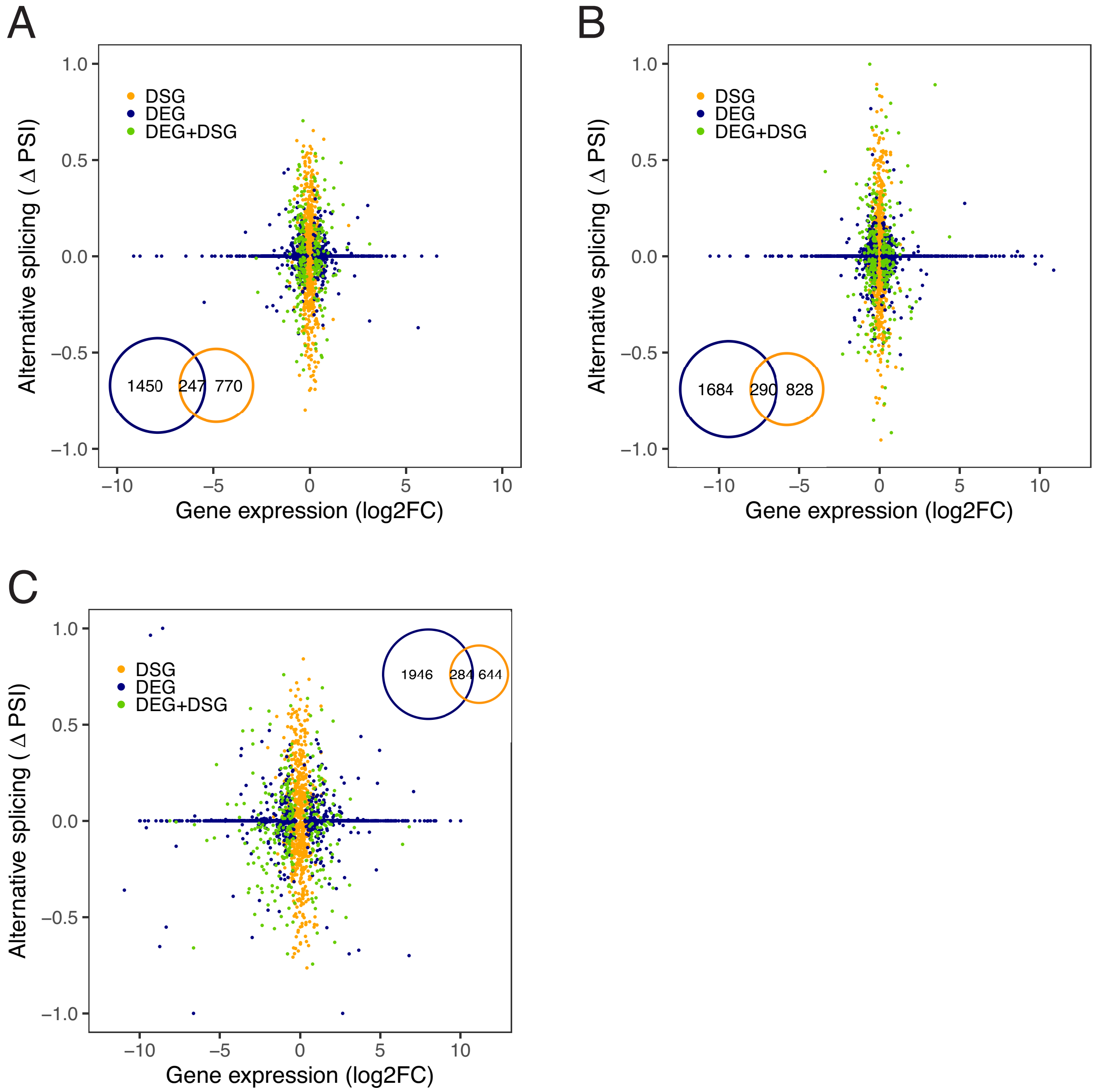

Supplementary Figure 1. Correlations between DEGs and DSGs in the seasonal forms during PP50, P15, and P50. The scatter plots show the magnitudes of gene expression differences (log2FC) of DEGs (padj<0.05), and inclusion level differences (ΔPSI) of DSGs (FDR<0.05), between seasonal forms (WS form vs DS form) during **(A)** PP50, **(B)** P15, and **(C)** P50. Venn plot shows the number of DEGs, DEGs, and genes belonging to both.

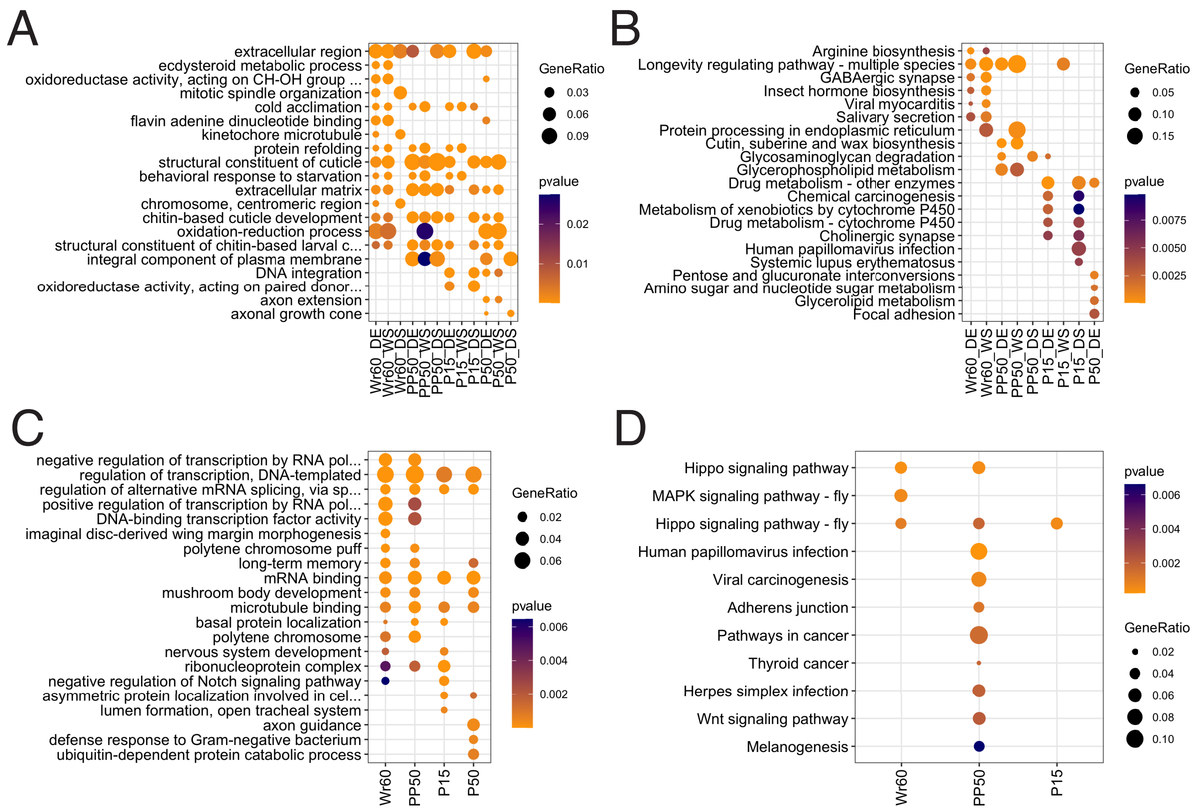

Supplementary Figure 2. Functional enrichment analysis of DEGs and DSGs between seasonal forms. (A-D) GO and KEGG functional enrichment analysis was performed for shortlisted DEGs (padj<0.05, |log2FC|>1) and DSGs (FDR<0.05, |ΔPSI|>0.1) between seasonal forms from each developmental timepoint. Top enriched functional terms were shown. **(A)** GO and **(B)** KEGG analysis for DEGs; **(C)** GO and **(D)** KEGG analysis for DSGs. For DEGs, each category was labeled as developmental stage_gene set. Gene set: DE, genes differentially expressed between seasonal forms; WS, genes up-regulated in the WS form; DS, genes up-regulated in the DS form. Dot color indicates p-values and dot size indicates gene ratio, which is the number of genes associated with the functional term divided by the total number of selected genes.

A

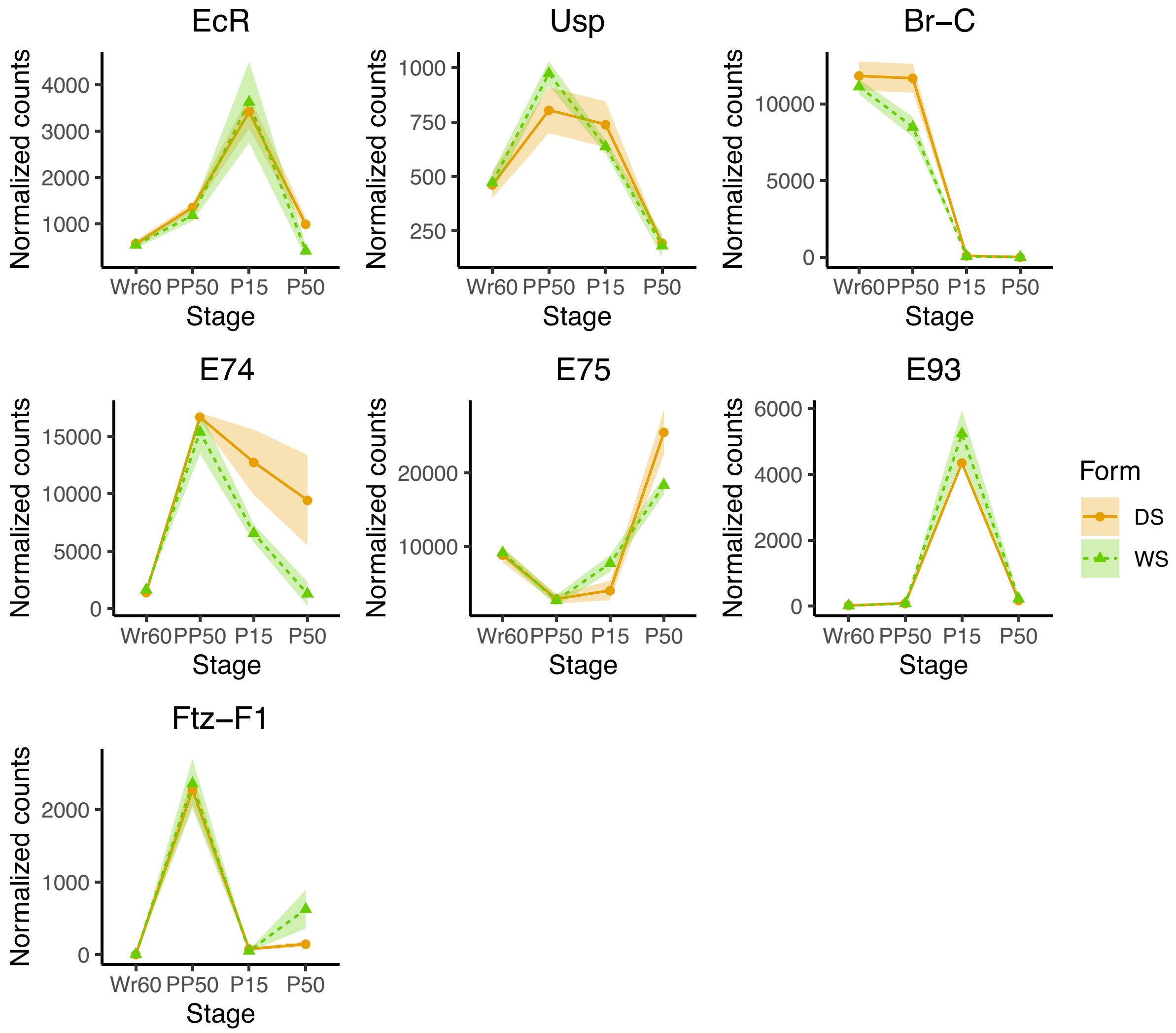

B

DSG	Type	Wr60 ΔPSI (DS-WS)	PP50 ΔPSI (DS-WS)
EcR	A3SS	-0.10	-0.11
Usp	A5SS	0.26	0.15
Br-C	SE	-0.04	-0.05
Ftz-F1	SE	0.00	-0.23

Supplementary Figure 3. Gene expression and splicing profiles of early 20E response genes. (A) Gene expression profiles (RNA-seq read counts normalized by DEseq2) of seven primary 20E response genes in the seasonal forms across four developmental timepoints. Shaded area indicates 95% confidential interval. **(B)** The figure highlights the differential splicing patterns of four of these factors between seasonal forms, during Wr60 and PP50, since these factors were also characterized as eyespot genes and the two stages span the larval 20E pulse, which primarily underlies eyespot size plasticity (Monteiro et al., 2015). Color denotes the relative ΔPSI values: Red, positive; Blue, negative.

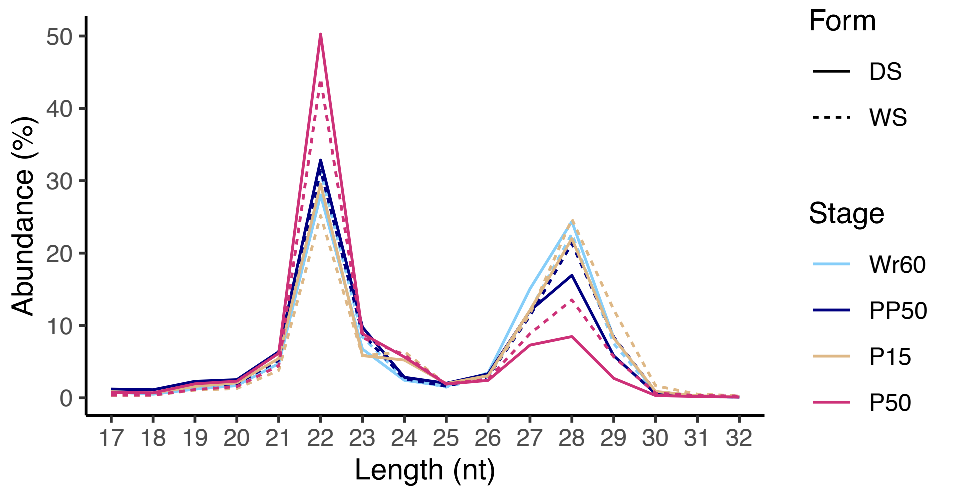

Supplementary Figure 4. Length distribution of small RNAs in *B. anynana* hindwings. The figure shows that after adaptor trimming, the majority of small RNAs in B. anynana hindwing tissue are 22nt and 28nt in length, typical length of miRNAs and piRNAs, respectively. The length distribution is consistent regardless of developmental stages or seasonal forms.

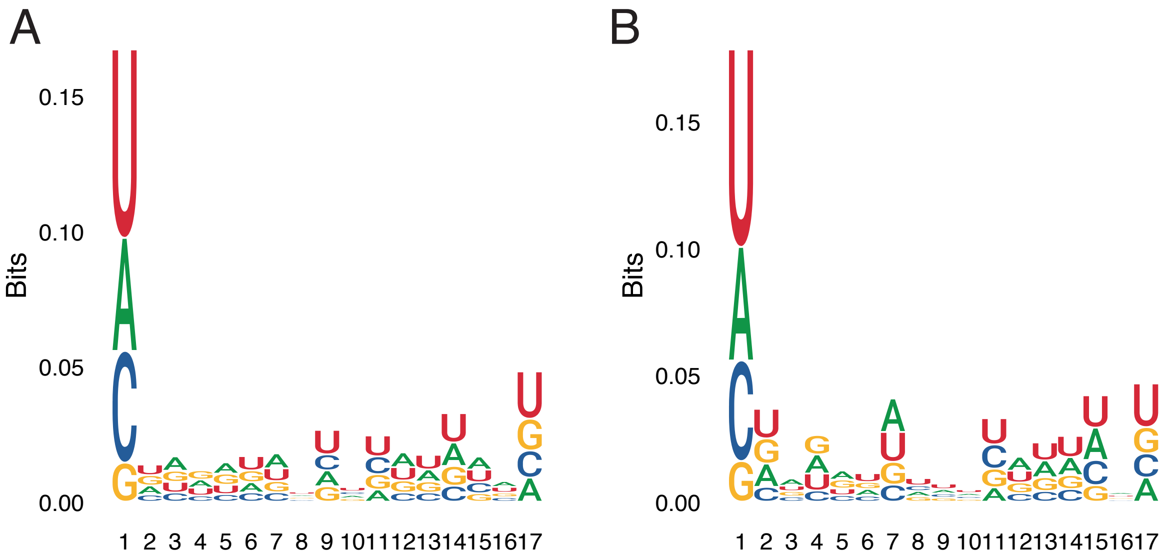

Supplementary Figure 5. Sequence characteristics of mature miRNAs annotated in *B. anynana* hindwings. Sequence logo was generated for **(A)** all miRNAs and **(B)** novel miRNAs characterized in *B. anynana* hindwing tissue, showing the nucleotide preference at each position across mature miRNA sequences. x axis represents position from the 5'-termini of mature miRNAs, y axis represents the information content (bit).

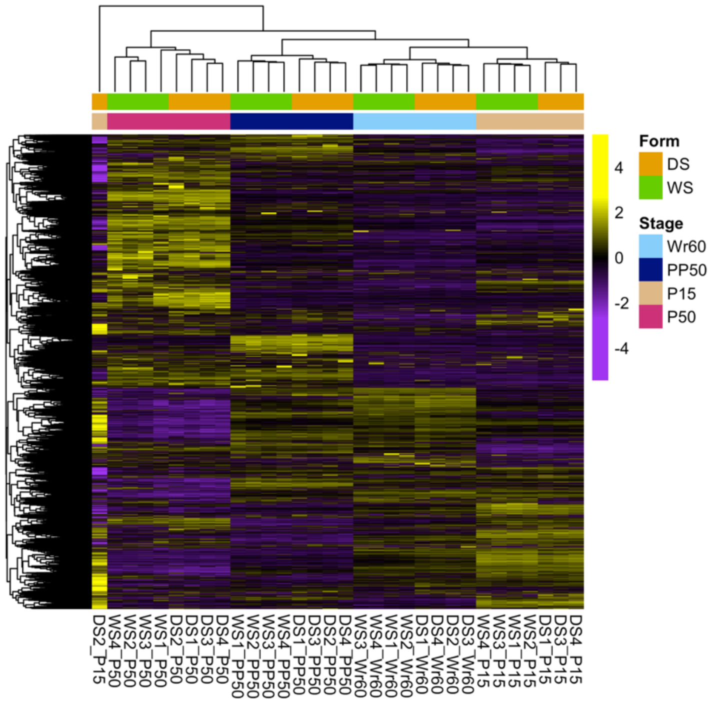

Supplementary Figure 6. Gene expression profile reveals that DS2_P15 is an outlier in the RNA-seq dataset. Hierarchical clustering heatmap was shown for the gene expression levels of all RNA-seq sequencing libraries, in which DS2_P15 appears as an outlier. This sample was excluded in all the bioinformatic analysis in this study. After removing this outlier, the sequencing libraries cluster according to developmental stages and seasonal forms, as shown in Fig, 2A lower panel.

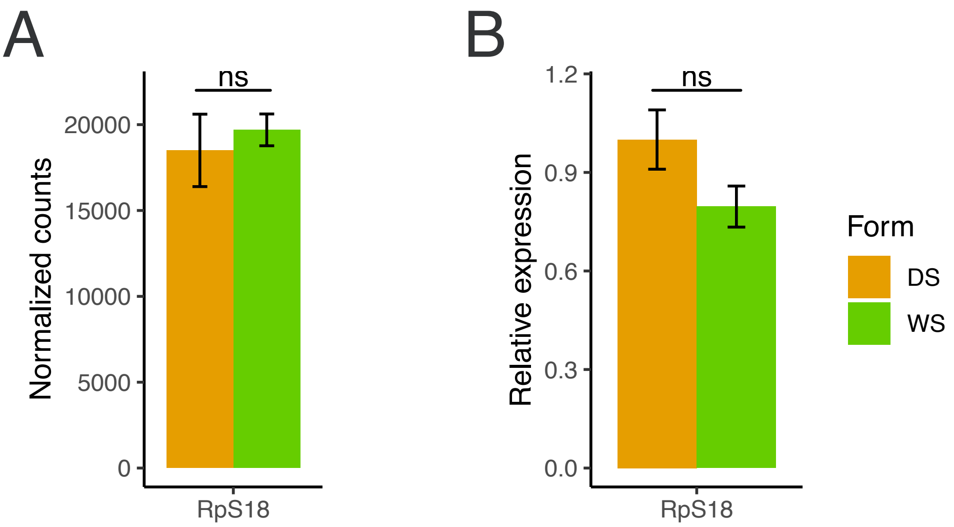

Supplementary Figure 7. Analysis of the stability of the housekeeping gene used in qPCR. (A) The housekeeping gene, *RpS18*, was chosen based on the stable expression between seasonal forms during Wr60 from the RNA-seq data. **(B)** Relative expression levels of *RpS18* were also confirmed to be equivalent between seasonal forms in the qPCR experiments, thus it could be used to normalize the expression of other *da* features during Wr60.
